# Comparison of concordance between chuna manual therapy diagnosis methods (palpation, X-ray, artificial intelligence program) in lumbar spine: An exploratory, cross-sectional, prospective observational study protocol

**DOI:** 10.1097/MD.0000000000028177

**Published:** 2021-12-23

**Authors:** Jin-Hyun Lee, Hyeon-Jun Woo, Jung-Han Lee, Joong-Il Kim, Jun-Su Jang, Young Cheol Na, Kwang-Ryeol Kim, Tae-Yong Park

**Affiliations:** a Institute for Integrative Medicine, Catholic Kwandong University International St. Mary's Hospital, Incheon, South Korea; b Department of Korean Rehabilitation Medicine, Wonkwang University College of Korean Medicine, Iksan, South Korea; c Digital Health Research Division, Korea Institute of Oriental Medicine, Daejeon, South Korea; d Department of Neurosurgery, Catholic Kwandong University International St. Mary's Hospital, Catholic Kwandong University College of Medicine, Incheon, South Korea.

**Keywords:** artificial intelligence, chuna manual therapy, diagnosis, manual medicine, radiology

## Abstract

**Introduction:**

Chuna manual therapy (CMT) is a type of manual medicine practiced by Korean medical doctors in South Korea. Spinal diagnosis in CMT uses a system that applies manual diagnostic and X-ray tests to detect specific vertebral malpositions, based on the relative alignment across vertebral bodies. Recently, artificial intelligence (AI) programs have been developed to assist in the radiological diagnosis of CMT using X-ray images. Nevertheless, a few clinical studies have reported on the concordance between diagnosticians, diagnostics methodologies, and the use of AI programs for diagnosing CMT. At present, the evidence to support CMT diagnosis is insufficient. This study thus aims to overcome such limitations by collecting and comparing CMT diagnostic data from experts and non-experts through manual diagnosis, X-ray test, and images obtained using an AI program. The study aims to search for CMT diagnosis methods with more outstanding rationality and consistency and to explore the potential use of AI-based CMT diagnosis programs.

**Methods/design:**

This study will be conducted as an exploratory, cross-sectional, prospective observational study that will recruit 100 non-specialist subjects. Each subject will submit a signed consent after the screening test and undergo L-spine standing AP & lateral X-ray imaging. Manual CMT diagnosis will be performed by 3 CMT experts according to the standard operation procedure (SOP). The X-ray images of the 100 subjects will subsequently be used to make the CMT radiological diagnoses according to the same SOP by the CMT expert group (n = 3) and CMT non-expert group (n = 3). Among the subjects, those in the non-expert group will receive another CMT radiological diagnosis with spinal data obtained using the AI program, approximately 1 month from after initial diagnosis.

Based on the collected diagnostic data, within- and between-group concordance levels will be assessed for each diagnostic method. The verified level of concordance will be used to test the potential use of CMT diagnostic method and CMT AI programs with high levels of rationality and consistency.

**Ethics and dissemination:**

This trial has received complete ethical approval from the Wonkwang University Korean Medicine Hospital (IRB 2021–8). We intend to submit the results of the trial to a peer-reviewed journal and/or conferences.

**Trial registration:**

https://cris.nih.go.kr/cris/search/detailSearch.do?search_lang=E&search_page=M&pageSize=10&page=undefined&seq=20613&status=5&seq_group=20613, Identifier: KCT0006707.

## 1 Introduction

Chuna manual therapy (CMT) is a method of manual therapy practiced by Korean medical doctors (KMDs) through the use of their hands, other body parts, or an assistive tool, such as the Chuna table, to apply an effective stimulation to the patient's body so as to treat a structural or functional problem.^[1]^ CMT was listed in the National Health Insurance Coverage in South Korea in 2019,^[2]^ and since then, valid effects have been reported for various musculoskeletal diseases.^[3,4]^

The spinal diagnosis system in CMT examines the upper vertebral bodies based on their relative position to the lower vertebral bodies, with standard kinetic viewpoints.^[5]^ Such CMT diagnoses most commonly apply a manual diagnosis that locates the spinal bony landmarks in the patient.^[1]^ Recently, to overcome the limitation of manual diagnosis in the evaluation of spinal alignment and improve diagnosis reproducibility, CMT diagnosis methods that use radiological devices, such as X-rays, have attracted increased interest as have other manual medicine methods, including osteopathy and chiropractic.^[6,7]^ In addition, recent attempts to measure spinal angles or diagnose spinal diseases have used artificial intelligence (AI) programs, such as conventional neural networks or deep learning.^[8]^ Positive results have also been reported for the AI programs to detect the lumbar vertebral landmarks in relation to CMT diagnosis.^[9]^

Nevertheless, the clinical evidence for such CMT-based spinal diagnosis methods is insufficient at present. Despite a number of studies that have investigated the concordance in manual medical diagnostic methods including CMT,^[10]^ the lack of adequate levels of evidence or reliability in manual medicine evaluation has been pointed out in systematic reviews of clinical studies on manual medical diagnostic methods regarding nonspecific lumbar pain.^[11]^ Another limitation is the insufficient number of clinical studies regarding CMT diagnosis compared to those regarding other diagnosis methods in manual medicine. Moreover, there is either a general or complete lack of studies on the concordance between diagnosticians, diagnostic devices, and the clinical use of AI programs in CMT diagnoses.

In this study, the concordance between diagnosticians and diagnostics used for each CMT diagnosis method commonly used in clinical practice will be compared to collect evidence to support the best use of CMT diagnosis methods. More effective CMT diagnosis methods will be explored, and the positive effects of CMT AI programs in clinical practice regarding diagnosis concordance and accuracy will be investigated, to provide basic data to test the potential use of AI programs in future clinical CMT applications.

## 2 Objectives

In this study, based on the CMT diagnosis of the lumbar spine, 4 diagnosis groups will be constructed;

1. CMT expert group (n = 3) for manual diagnosis;
2. CMT expert group (n = 3) for radiological diagnosis;
3. CMT non-expert group (n = 3) for X-ray radiological diagnosis;
4. CMT non-expert group (n = 3) for AI-based X-ray radiological diagnosis.

The results will be collected for the specific diagnosis performed on the study subjects. Within- and between-group concordance levels will be subsequently compared to search for clinically effective CMT diagnosis methods. The potential use of the AI-based CMT diagnosis program will also be verified.

## 3 Methods

### 3.1 Trial registration

This study has been registered in the Cris.nih.go.kr (Trial registration number: KCT0006707; protocol version 1.2; https://cris.nih.go.kr/cris/search/detailSearch.do?search_lang=E&search_page=M&pageSize=10&page=undefined&seq=20613&status=5&seq_group=20613).

### 3.2 Study design

This study is an exploratory, cross-sectional, prospective observational study. The number of subjects to be recruited is 100, and data from patient enrolment, manual diagnosis by CMT experts, to X-ray imaging are to be collected at the Wonkwang University Korean Medicine Hospital, Iksan, South Korea. CMT diagnostic data obtained from X-ray images are to be collected at the Catholic Kwandong University International St. Mary's Hospital, Incheon, South Korea, where CMT expert and non-expert groups are defined.

**Figure 1:**
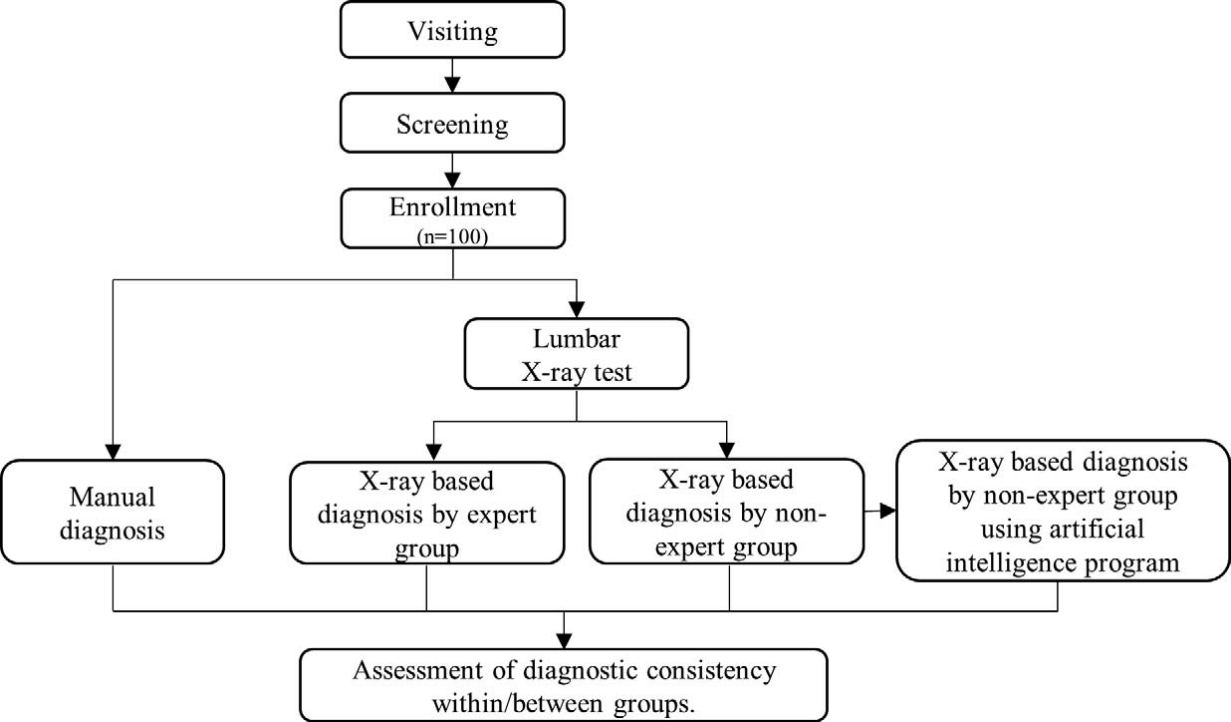
Flow chart of the study.

The study flow chart and its details are presented in Figure 1. The recruited subjects will be provided with an adequate and detailed explanation on the study, after which they will submit signed consent. The enrolled subjects will visit the study center at least twice. The subjects will undergo screening (visit 1), and after submitting consent, they will be examined to verify whether they are participating in another clinical study and identify their demographics and basic physical data (height, weight, body mass index, and vital signs). Inclusion and exclusion criteria will also be applied. The finally-selected subjects will undergo subsequent steps (visit 2), and after being assigned with an individual registration number, they will receive a basic physical examination, an evaluation of lumbar pain, and an X-ray test (L-spine standing AP & Lateral). Next, the same subjects will undergo a manual CMT diagnosis by each of the 3 CMT experts, and the relevant information will be documented in the case report form (CRF). All subjects will undergo identical diagnoses, without being divided into specific groups. The steps at visit 1 and visit 2 may be performed on identical dates, and of the steps at visit 2, the X-ray test and CMT manual diagnosis (or palpation) may not be performed within a single day, in which case the respective subject will make a third visit within a week from visit 2. After completing all scheduled visits, the subjects will no longer be required to make further visits. The CMT manual diagnosis and X-ray test will be performed once for each subject.

The X-ray images of the 100 subjects collected at the Wonkwang University Korean Medicine Hospital will be anonymized in a DICOM format that removes the subject identification data and then transferred to the Catholic Kwandong University International St. Mary's Hospital. Using these images, the CMT diagnosis will be performed independently by CMT expert and non-expert groups (3 individuals in each group). To prevent learning effects, the CMT non-expert group will perform the radiological diagnosis once more after around a month, using the same X-ray data, but this time, with the assistance of an AI program to define the relative position of the lumbar vertebral bodies for CMT diagnosis.

The investigators responsible for the clinical study, who participate in the process of diagnosis, will comply with the same standard operation procedure (SOP) for each diagnosis method. The diagnosis results collected in this study will be analyzed for within- and between-group concordance levels per diagnosis method.

### 3.3 Inclusion and exclusion criteria

Inclusion criteria

1. Adult male or female aged 20 to 60 years
2. Ability to understand the study protocol and agrees to participation, and who voluntarily signs the institute review board (IRB)-approved consent form
3. No communication problems during the physical examination and X-ray test

Exclusion criteria

1. Past trauma or surgery that may induce a structural problem in the lumbar spine (e.g., osteoporosis, fracture, and lumbar spine surgery)
2. A history of disease that may induce a structural change in the lumbar spine (e.g., congenital spinal deformity, ankylosing spondylitis, idiopathic scoliosis, and tumor)
3. An obesity factor of mid to high severity based on BMI (>30 kg/m^2^) that prevents manual spinal diagnosis
4. A history of psychotic disorder, alcohol addiction, or drug addiction
5. Pregnant mothers or women planning pregnancy
6. Other individuals deemed unsuitable for the study by the principal investigator

### 3.4 Sample size calculation

As this study is an exploratory clinical study, it was deemed unnecessary to calculate the sample size using the standard formula. The guideline of the evaluation of clinical effectiveness of AI-based medical devices (Ministry of Food and Drug Safety, South Korea, 2019) suggests that, for cases where the sample size cannot be estimated using a statistical method, the required number of subjects may be calculated in reference to relevant data (e.g., a published clinical study, etc.).^[12]^ The estimated number of subjects was 100 based on a review of previous studies related to this 1;

1. a study by Lee et al that analyzed the concordance between the CMT manual diagnosis and radiological diagnosis (n = 22)^[13]^;
2. a study that compared the results of expert/non-expert/AI diagnosis of spinal alignment using X-ray images (n = 35)^[14]^;
3. a study that compared the X-ray angles between experts and non-experts to test a program that automatically detects the Cobb's angle in scoliosis.^[15]^

### 3.5 CMT diagnosis panel

#### 3.5.1 CMT expert group

The CMT expert group contains 3 KMD members who satisfy at least 1 item of the following criteria:

1. A specialist with the certification of Korean Medicine Rehabilitation
2. A certified lecturer or educational board member of the Korean Society of Chuna Manual Medicine for Spine and Nerves (KSCMMSN)
3. An individual who completed the regular workshop of the KSCMMSN
4. An individual with a published study regarding the spinal CMT diagnosis

#### 3.5.2 CMT non-expert group

The CMT non-expert group contains 3 KMD members who perform CMT in clinical practice but do not satisfy the aforementioned criteria of the CMT expert group.

### 3.6 CMT diagnosis methods in this study

#### 3.6.1 Overview of CMT diagnosis

**Figure 2:**
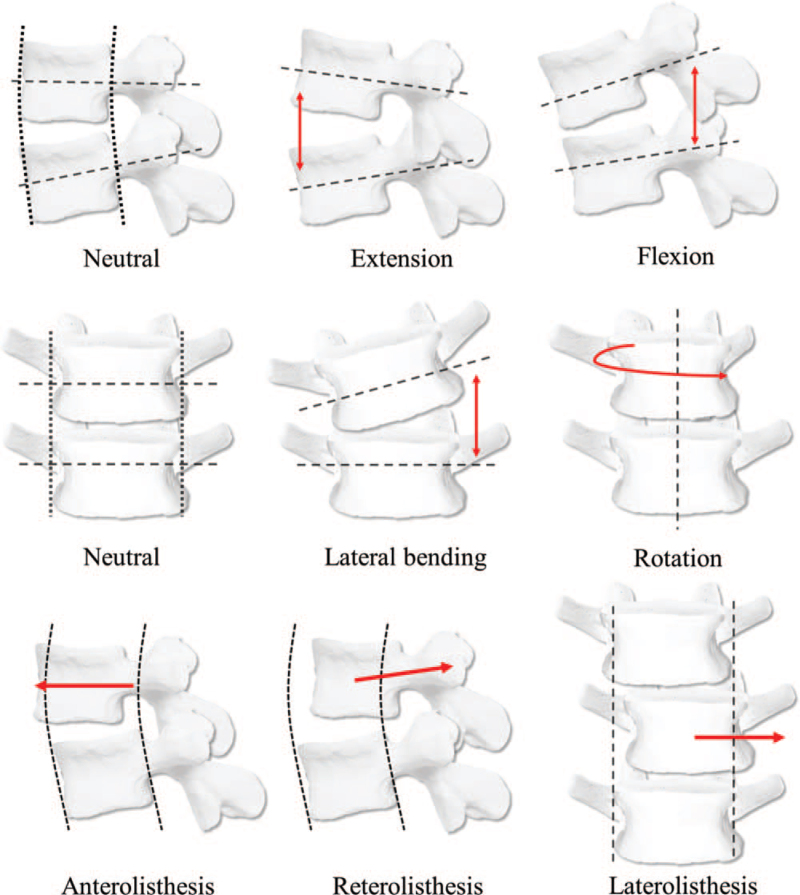
Classification system of spinal alignment diagnosis in Chuna manual therapy.

In CMT diagnoses, spinal alignment is assessed based on the relative 3D motion and alignment of the upper vertebral bodies to the lower vertebral bodies along 3 axes; vertical, anterior-posterior, and medial-lateral.^[5]^ With the neutral position as the base, malpositions related to the flexion and extension, right/left rotation, and right/left lateral bending are examined. Each malposition may appear on its own or in combination on a single lumbar level. In addition, for cases where axial alignment is disrupted or upper vertebral bodies show a slip in relation to the lower vertebral bodies, the diagnosis is *–lithesis*; anterolisthesis, retrolisthesis, or laterolisthesis, according to the direction of the slip (Fig. 2).

A more refined spinal alignment assessment is performed based on3 broad categories:

1. Any abnormality regarding CMT spinal alignment;
2. The position of the lumbar level (multiple positions may be recorded) for the diagnosis of an abnormal CMT spinal alignment;
3. The type of abnormality for the CMT lumbar level with a problem.

The panel diagnosis is to be based on answering questions under these 3 categories, while each is completely separate from the other, to prevent interference or interactions between investigator responses.

#### 3.6.2 Palpation (manual) diagnosis

The CMT experts performing the manual diagnosis will follow the same SOP described below, which combines key contents from CMT textbooks and published articles for a manual diagnosis of spinal alignment.^[1,16]^

### 3.7 Assessment of flexion/extension and rotation lateral bending malpositions

The subjects are guided to maintain the neutral position without flexion or extension of the spine, while the overall transverse and spinous processes are manually examined from the sacrum base to the lumbar spine to determine the positional characteristics across each segment.

The subjects are then guided to show the flexion, extension, left/right lateral bending, and left/right rotation motions, and as in 1), the transverse process of each motion is manually examined to assess the changes to the alignment at specific segments in contrast to the neutral position.

The spinal level with flexion or extension malposition is determined based on the reduced motion in comparison to the normal lumbar segments in the respective posture. Also, the spinal segments with left/right rotation or lateral bending malposition are determined based on reduced motion, in comparison to normal lumbar segments in the rotation or lateral bending of the opposite direction.

At the completion of all tests, the investigator records the defined type in the CRF.

#### 3.7.1 Assessment of anterolisthesis, laterolisthesis, and retrolisthesis^[17]^

The subjects are guided to maintain the neutral position without flexion or extension of the spine.

The investigator stays behind the subject to monitor and assess the alignment of the spinous process of the lumbar spine and the symmetry of skin folds around the spine.

The investigator then manually examines the lumbar spine along the spinous process to assess any areas of dents or protrusions or bending towards a side away from the sagittal axis. If a dent is found upon the manual examination of the spinous process, the diagnosis is anterolisthesis. If a posterior protrusion is found, the diagnosis is retrolisthesis. If the spinous process is bent away from the central line of the spine despite the lack of abnormalities in 1), the diagnosis is laterolisthesis.

At the completion of all tests, the investigator records the defined type in the CRF.

#### 3.7.2 X-ray test

The radiological diagnosis using X-ray images is performed by 2 groups; the CMT expert group (n = 3) and the CMT non-expert group (n = 3). The 2 groups receive anonymized lumbar X-ray AP and lateral view data in a file format, and, according to the same SOP used in prior CMT radiological diagnosis studies, the diagnosis is made based on the positions of vertebral bodies and the relative positions of surrounding structures.^[6]^ The spinal radiological examination results are based on the same categorization system and recorded in the same CRF documents used in manual diagnosis.

#### 3.7.3 X-ray test using an AI program

**Figure 3:**
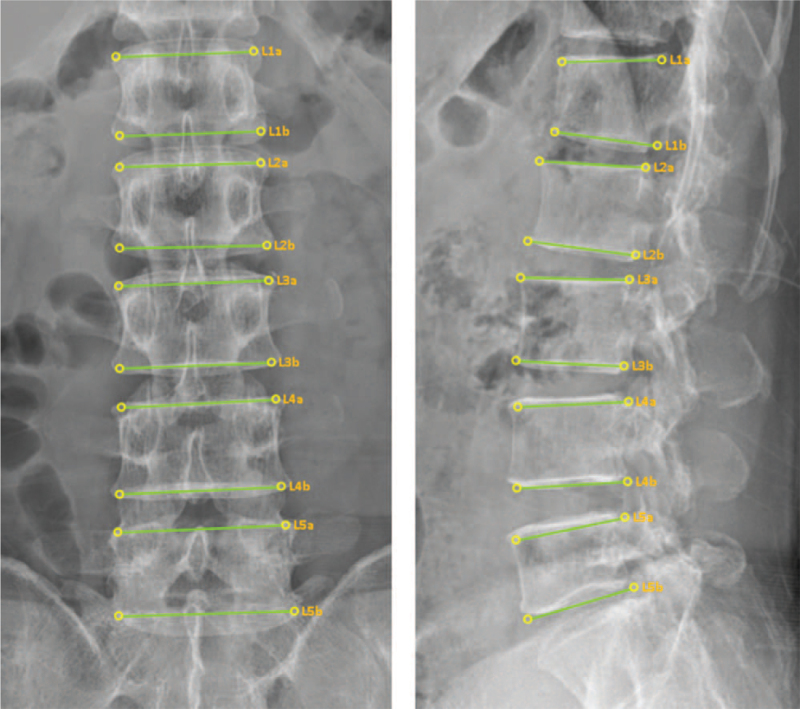
Example of the feature point locations suggested by the AI program.

An artificial intelligence (AI) program based on convolutional neural networks will be used in this study. CNNs are widely used in medical image analysis field due to their excellent performance. The AI program will be trained to automatically detect the vertebral feature points of the lumbar spine. The detailed program structure is described in reference 11. The AI program suggests the locations of 4 feature points per a vertebra. Figure 3 shows an example of the feature point locations suggested by the AI program. The performance of the AI program was reported with a success rate of 99.7% and a position error of 4.54 ± 3.00 pixels.^[9]^

The CMT non-experts (n = 3) who perform the radiological diagnosis will once more be provided with the X-ray images, which will be identical to the previous ones, around a month after the initial diagnosis, together with an AI program. They will then use the AI program to perform a CMT radiological diagnosis based on the reference points suggested by the AI program, to assist with the detection of the positions of vertebral bodies in the lumbar spine. The results will all be recorded in the same CRF used in the manual and radiological diagnoses.

### 3.8 Recruitment, randomization, blinding, and non-blinding

The subject recruitment ad will be posted on the hospital notice boards and websites of Wonkwang University Korean Medicine Hospital. All ads to be used in this study will first be approved by the IRB.

This study is an exploratory, cross-sectional, prospective observational study that does not involve randomization or a process related to blinding. However, to prevent evaluation bias, an independent investigator will perform the subject screening, collection of demographic data, and analysis of baseline health and medical history. All investigators in the CMT diagnosis will also be assigned an independent space to perform the evaluations, while any unnecessary talks or contact among different investigators or with subjects will be prevented during the evaluations, so that the investigators cannot acquire knowledge of the diagnosis results of other investigators. The investigators that perform the radiological diagnosis will solely use anonymized X-rays images, while appropriate measures will be applied to prevent the investigators from sharing the diagnosis data among themselves.

### 3.9 Study outcomes

Within-group concordance between diagnosticians per diagnosis method

For the within- and between-group comparisons of the CMT diagnosis results, the following categories are set to evaluate the concordance. Through this, the concordance between diagnosticians can be compared for each diagnosis method, to determine the methods with higher concordance levels.

1. Concordance regarding spinal alignment abnormalities
2. To evaluate concordance in relation to spinal alignment abnormality, an individual must show at least 1 single lumbar level abnormality.
3. Concordance regarding the lumbar level with a spinal alignment abnormality
4. To evaluate the concordance in relation to the lumbar level marked for spinal alignment abnormality.
5. Concordance regarding the type of spinal alignment abnormality

To evaluate the concordance in relation to the type of spinal alignment abnormality.

#### 3.9.1 Between-group concordance per diagnosis method

For the aforementioned spinal alignment abnormality, the lumbar level with the spinal alignment abnormality, and the type of spinal alignment abnormality, the diagnosis concordance is evaluated as follows.

1. Concordance regarding the manual and radiological diagnoses by the CMT expert group
2. Concordance regarding the manual diagnosis by the CMT expert and CMT non-expert groups
3. Concordance regarding the manual diagnosis by the CMT non-expert group and that using an AI program
4. Concordance regarding the AI-based diagnosis by the CMT non-expert group and the radiological diagnosis by the CMT expert group

#### 3.9.2 Monitoring of early termination, dropout, safety, and adverse events

As no specific intervention will be applied in this exploratory study for diagnostic purposes, no additional analysis will be carried out on early termination, dropout, safety, or adverse events.

### 3.10 Statistical analysis

#### 3.10.1 Demographic data and subject characteristics prior to treatment

The subject demographics and base characteristics will be analyzed using descriptive statistics. For continuous variables, the number of observed values, mean, standard deviation, median, and 25% and 75% percentiles will be used. For categorical variables, the frequency and percentage will be used.

#### 3.10.2 Concordance analysis

To analyze the concordance levels for the results obtained by the 3 investigators in each group, the Fleiss’ Kappa evaluation will be used to determine the between-diagnostician concordance. If necessary, the Cohen's Kappa evaluation will be used to determine the between-investigator/group levels. For missing values, the Akaike information criterion will be applied.

### 3.11 Data collection, access, management, and monitoring

All subject identification data will be anonymized using initials, and the data collected in this study will be recorded immediately in the relevant documents and the CRF. All subjects will maintain confidentiality of the results. All documents related to this study will be stored in a secure, isolated space according to the IRB regulations at Wonkwang University Korean Medicine Hospital. The data obtained in this study will be anonymized, and data access will be restricted to the investigators participating in the study and those included in the consent form, after approval from the principal investigator. All participating investigators will maintain confidentiality of the clinical outcomes.

Data management will be performed according to the clinical trial regulations at Wonkwang University Korean Medicine Hospital. An independent monitoring will also be performed according to hospital regulations at Wonkwang University Korean Medicine Hospital, to ensure the study is conducted in compliance with the clinical study protocol and the Korea Good Clinical Practice. During the monitoring period, the CRF will be reviewed based on a comparison with the evidential documents, to ensure the records are clear and complete.

### 3.12 Ethics and Dissemination

#### 3.12.1 Research ethics approval

This trial has received complete ethical approval from the Ethics Committee of Wonkwang University Korean Medicine Hospital (IRB 2021–8).

#### 3.12.2 Protocol amendments

If protocol amendments are necessary, the relevant contents will be discussed with the principal investigator. If the clinical study protocol requires amendments, IRB approval will first be obtained. However, if in an emergency case occurs during the study period that demands the immediate treatment of the subject, the protocol may be altered, and the change will be reported to the IRB afterwards.

#### 3.12.3 Consent

The recruited subjects will be given an adequate and detailed explanation of the study by an investigator who will not participate in the diagnostic process. If a subject has a question regarding the study flow, a request for adequate explanation can be made to the investigators participating in the study. The subjects who agree to participation in the study will subsequently submit a signed consent form.

#### 3.12.4 Confidentially

All subject identification data will be anonymized using initials or symbols. All contents related to the results of the clinical study will be privately maintained by each diagnostician, who will also store the signed consent from the subjects and create a list of data that contains the subject's name, identity, and registration number for future reference.

#### 3.12.5 Declaration of interests

None.

#### 3.12.6 Rescue therapy and post-trial care

This study will be conducted as an exploratory, cross-sectional, prospective observational study to collect the diagnostic results. As it does not involve clinical interventions, no additional regulations will be developed regarding post-trial care. However, if an emergency occurs during data collection, appropriate measures will be taken in accordance with the emergency protocols at Wonkwang University Korean Medicine Hospital and in compliance with the Korea Good Clinical Practice guidelines and the revised version of the Declaration of Helsinki, to ensure ethical and scientific considerations.

#### 3.12.7 Dissemination policy

The findings in this study will be disseminated through peer-review journals and/or conference presentations.

## 4 Discussion

The purpose of this clinical study is to compare and collect the evidence of the diagnostic concordance between diagnosticians and the diagnostics most widely used and well-known in CMT. The study has the following advantages: First, among the studies published so far regarding CMT diagnosis, this study proposes a design based on the largest number of cases and diagnosticians. Previous studies either had a very small number of cases or relied solely on a single diagnostician to pose a difficulty in defining sufficient rational evidence of the diagnosis outcomes.^[13]^ Thus, it is anticipated that the present study will provide the basic data for establishing a higher level of evidence in the CMT diagnostic field.

Second, most previous studies on manual diagnoses obtained the diagnoses based on the personal experience of the diagnostician, without an established SOP and compared the outcomes. On the contrary, the diagnosis in this study is performed according to an SOP that was produced using the contents of regular educational courses conducted by all Korean Medicine colleges in South Korea and by the KSCMMSN. The type of diagnoses are also specified based on the respective spinal abnormality, lumbar level with abnormality, and type of abnormality, to allow an in-depth analysis of the items with a high level of diagnostic concordance and those with a low level of concordance. Through this process, the rational diagnostic capabilities of current regular educational courses can be verified. The study will also provide basic data for developing further diagnostics or educational methods for items with a high error rate. In addition, comparing the manual diagnosis results between the CMT expert and non-expert groups is likely to induce additional discussions on methods for improving diagnoses by non-expert groups.

Lastly, the significance of this study lies in its being the first clinical study to apply an AI program in CMT as well as in the field of manual medicine, to the best of our knowledge. For CMT radiological diagnoses assisted by an AI program, the potential use of AI-based CMT diagnosis programs in clinical practice can be tested by determining whether the methodology results in an improvement in diagnostic concordance by CMT non-experts compared to CMT experts.

While this is an exploratory study, its findings are anticipated to provide basic data for identifying CMT diagnosis methods with high levels of rationality and evidence and in the development of CMT AI programs. Furthermore, the study results will lead to additional clinical studies that use CMT diagnosis methods and AI programs with reinforced evidence.

## Author contributions

**Conceptualization:** Jin-Hyun Lee, Joong-il Kim, Jun-Su Jang, Young Cheol Na, Kwang-Ryeol Kim, Tae-Yong Park.

**Data curation:** Hyeon-Jun Woo, Jung-Han Lee, Jun-Su Jang, Young Cheol Na, Kwang-Ryeol Kim, Tae-Yong Park.

**Funding acquisition:** Jin-Hyun Lee.

**Investigation:** Hyeon-Jun Woo, Jung-Han Lee.

**Methodology:** Jun-Su Jang, Young Cheol Na, Kwang-Ryeol Kim, Tae-Yong Park.

**Project administration:** Hyeon-Jun Woo, Jung-Han Lee, Tae-Yong Park.

**Resources:** Hyeon-Jun Woo, Jung-Han Lee.

**Software:** Joong-il Kim, Jun-Su Jang.

**Supervision:** Jung-Han Lee.

**Writing - original draft:** Jin-Hyun Lee, Tae-Yong Park.

**Writing - review & editing:** Jin-Hyun Lee, Tae-Yong Park.

## References

[1] Korean society of Chuna manual medicine for spine and nerves. Chuna manual medicine [Korean]. 2.5 ed. Seoul, South Korea: Korean society of Chuna manual medicine for spine and nerves; 2017:4, 65–74.

[2] Yu J Shin BC Kim H. The process of National Health Insurance coverage for chuna manual therapy in Korea: a qualitative study. Integr Med Res 2022;11:100746. 34277346 10.1016/j.imr.2021.100746 PMC8267429

[3] Lee J Cho JH Kim KW. Chuna manual therapy versus usual care for patients with nonspecific chronic neck pain: a randomized clinical trial. JAMA 2021;4:e2113757. 10.1001/jamanetworkopen.2021.13757 PMC8280970 34259850

[4] Lee NW Kim GH Heo I. Chuna (or Tuina) manual therapy for musculoskeletal disorders: a systematic review and meta-analysis of randomized controlled trials. Evid Based Complement Alternat Med 2017;2017:8218139. 29441114 10.1155/2017/8218139 PMC5758860

[5] Shin BC Cho HW Hwang EH Sul JU Shin MS Nam HW. An literatural study of listing system of spinal subluxation [Korean]. J Korea CHUNA Manual Med Spine Nerves 2011;6:141–8.

[6] Lee J-H Kim C-G Jo D-C. Diagnostic X-ray from the perspective of chuna manual medicine, based on the listing system of spinal and pelvic subluxation [Korean]. J Korea CHUNA Manual Med Spine Nerves 2014;9:01–14.

[7] Kim TG Gi Y Yang KJ. The implications of X-ray use in chuna manual therapy from the viewpoint of Korean Medicine Doctors [Korean]. J Acupunct Res 2018;35:108–14.

[8] Galbusera F Casaroli G Bassani T. Artificial intelligence and machine learning in spine research. JOR Spine 2019;2:e1044. 31463458 10.1002/jsp2.1044 PMC6686793

[9] Jang JS. A study on lumbar vertebrae landmark detection using convolutional neural networks [Korean]. J Converg Inf Technol 2020;9:263–72.

[10] van Trijffel E Lindeboom R Bossuyt PM. Indicating spinal joint mobilisations or manipulations in patients with neck or low-back pain: protocol of an inter-examiner reliability study among manual therapists. Chiropr Man Therap 2014;22:22. 10.1186/2045-709X-22-22 PMC4074830 24982754

[11] May S Littlewood C Bishop A. Reliability of procedures used in the physical examination of non-specific low back pain: a systematic review. J Physiother 2006;52:91–102. 10.1016/s0004-9514(06)70044-7 16764546

[12] Guidelines for Clinical Efficacy Evaluation of Artificial Intelligence [AI] Based Medical Devices [Korean]. Cheongju-si, South Korea: Medical Device Review Department, Food and Drug Administration, Ministry of Food and Drug Safety; 2019:6-7.

[13] Lee JM Koog JH Choi B Jeong HA Hong SY. The comparative study between leg length analysis and X-ray on diagnosis of pelvic malpositions - according to positions and valuation bases - [Korean]. J Korea CHUNA Manual Med Spine Nerves 2010;5:169–80.

[14] Horng MH Kuok CP Fu MJ Lin CJ Sun YN. Cobb angle measurement of spine from X-ray images using convolutional neural network. Comput Math Methods Med 2019;2019:6357171. 30996731 10.1155/2019/6357171 PMC6399566

[15] Zhang J Li H Lv L Zhang Y. Computer-aided Cobb measurement based on automatic detection of vertebral slopes using deep neural network. Int J Biomed Imaging 2017;2017:9083916. 29118806 10.1155/2017/9083916 PMC5651147

[16] DeStefano LA. Greenman's Principles of Manual Medicine. 5th ed. 2017;Philadelphia: Wolters Kluwer, 288–313.

[17] Ahn K Jhun HJ. New physical examination tests for lumbar spondylolisthesis and instability: low midline sill sign and interspinous gap change during lumbar flexion-extension motion. BMC Musculoskelet Disord 2015;16:97. 25896607 10.1186/s12891-015-0551-0 PMC4419388

